# First report on the molecular phylogenetics and population genetics of *Aedes aegypti* in Iran

**DOI:** 10.1186/s13071-024-06138-3

**Published:** 2024-02-01

**Authors:** Azim Paksa, Kourosh Azizi, Saideh Yousefi, Sorna Dabaghmanesh, Saeed Shahabi, Alireza Sanei-Dehkordi

**Affiliations:** 1 Department of Biology and Control of Disease Vectors, School of Health, Shiraz University of Medical Sciences, Shiraz, Iran; 2 Sirjan School of Medical Sciences, Sirjan, Iran; 3 Infectious and Tropical Diseases Research Center, Hormozgan Health Institute, Hormozgan University of Medical Sciences, Bandar Abbas, Iran; 4 Department of Biology and Control of Disease Vectors, Faculty of Health, Hormozgan University of Medical Sciences, Bandar Abbas, Iran

**Keywords:** *Aedes aegypti*, mtDNA-COI gene, Genetic diversity, Haplotype, Iran

## Abstract

**Background:**

*Aedes aegypti*, the primary vector of various human arboviral diseases, is a significant public health threat. *Aedes aegypti* was detected in Iran in 2018, in Hormozgan province, but comprehensive information regarding its genetic diversity and origin within the country remains scarce. This study aimed to determine the origin and genetic diversity of *Ae. aegypti* in southern Iran.

**Methods:**

*Aedes aegypti* mosquitoes were collected from Bandar Abbas City, Hormozgan Province, southern Iran, between May and July 2022. Specimens were morphologically identified. Origin and assess genetic diversity were assessed based on the mitochondrial DNA-encoded cytochrome *c* oxidase subunit I (mtDNA-COI) gene.

**Results:**

BLAST (basic local alignment search tool) analysis confirmed the accuracy of the morphological identification of all specimens as *Ae. aegypti*, with 100% similarity to GenBank sequences. Calculated variance and haplotype diversity were 0.502 and 0.00157, respectively. Among the 604 examined nucleotide sequences, only a single site was non-synonymous. Total nucleotide diversity and average pairwise nucleotides were determined as 0.00083 and 0.502, respectively. Fu and Li's D test values were not statistically significant. Strobeck’s S statistic value was 0.487, and Tajima’s D value was 1.53395; both were not statistically significant (*P* > 0.10).

**Conclusions:**

Phylogenetic analysis revealed two distinct clades with minimal nucleotide differences and low haplotype diversity, suggesting the recent establishment of *Ae. Aegypti* in the southern region of Iran. The phylogenetic analysis also indicated an association between *Ae. aegypti* populations and mosquitoes from Saudi Arabia and Pakistan.

**Graphical Abstract:**

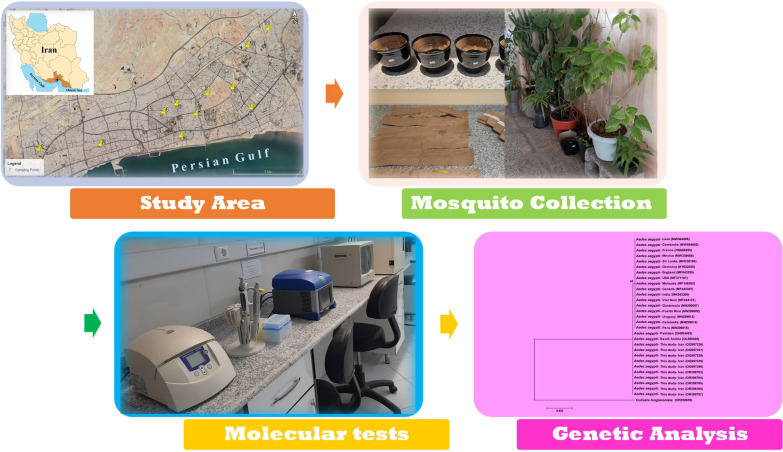

## Background

Female *Aedes aegypti* mosquitoes blood-feed on mammalian hosts, but they exhibit a strong preference for humans over alternative hosts, making them a significant vector of medically important arboviral pathogens, such as dengue fever virus (DENV), Zika virus (ZIKV), chikungunya (CHIKV) and West Nile virus (WNV) [1–4]. The widespread prevalence of *Ae. aegypti* across diverse continents therefore represents a significant health threat to millions of people around the world [5, 6]. The close association between *Ae. aegypti* and humans, particularly in tropical and subtropical regions, has resulted in this species being a primary vector for the indiscriminate spread of arbovirus diseases.

*Aedes aegypti* originated in Africa and likely dispersed to other continents through sea trade and air travel [7]. Genetic analyses indicate notable distinctions between African *Ae. aegypti* mosquito populations and those found on other continents. Two subspecies of *Ae. aegypti* are formally recognized: *Ae. aegypti formosus,* which has a relatively darker body color, is primarily found in Africa, and *Ae. aegypti aegypti*, which has a relatively lighter body color, is prevalent on the continents of Asia, Europe and the Americas [8, 9]. *Aedes aegypti aegypti* has an affinity for human blood and has been widely disseminated in tropical and subtropical regions globally by human activity [10], while the ancestral forms of the sub-Saharan African species *Ae. aegypti formosus* have been discovered in forested areas where they primarily feed on small mammals [11]. The species breeds most commonly in artificial containers, but also in tree holes, and is predominantly found indoors.

The movement of populations, goods and animals between Iran and other countries where * Aedes*-borne diseases are persistent has provided numerous opportunities for the transmission and spread of arboviral diseases caused by DENV, CHIKV and WNV within Iran [12, 13]. In Iran, the seroprevalence of CHIKV in the human population was detected in earlier studies, providing evidence of mosquito infection with CHIKV originating from Iran [14, 15]. DENV is considered to be the most significant arbovirus transmitted among humans by *Aedes* mosquitoes [10]. The majority of human cases have been documented in southeastern Iran, near the border with Pakistan, with no evidence of viral RNA presence in mosquitoes [15]. To date, there is no documented evidence for the occurrences of ZIKV and YFV in Iran [16]. In contrast, WNV transmission is frequently reported across various regions in Iran [17, 18]. Previous studies have indicated that both humans and horses serve as common vertebrate hosts for WNV in Iran [19, 20]. Notably, a study identified three encephalitis patients who tested positive for WNV, with 1.3% of humans and 2.8% of horses found to have positive serological sera [21]. WNV was also identified in mosquitoes such as *Aedes caspius* and *Culex pipiens* found in both northern and southern regions of Iran [13, 21, 22].

Molecular methods are currently widely used to investigate the origin and pathways of alien species, gene flow patterns and the genetic composition of specific populations [23, 24]. Genetic diversity within mosquito populations, notably *Ae. aegypti*, remains critical in terms of their adaptation to environmental conditions [25]. Accurate insights into the genetic diversity of *Ae. aegypti* populations in specific regions is essential for devising mosquito control strategies. Consequently, genetic diversity studies on vector mosquito populations, particularly *Ae. aegypti* populations, can provide valuable insights into the pathways of pathogen transmission, leading to more effective disease vector control strategies and providing crucial information for application in control measures against these diseases. Such studies also enable the identification of distinct populations in specific areas, offering valuable information on geographic distribution, biological characteristics, population genetic structures over time and assessments of reproductive isolation [26].

Although the genetic diversity of mosquitoes, especially *Ae. aegypti*, has been extensively studied globally [10, 27–35], limited information is available on the distribution and genetic diversity of this species in Iran [36–40]. Hence, the aim of the present study was to investigate the origin, genetic diversity and phylogeny of *Ae. aegypti* collected from southern Iran. The findings of this study are crucial for comprehending and effectively managing the *Ae. aegypti* population in Iran and will facilitate the development of better recognition and control strategies against this species in Iran.

## Methods

### Study area

Bandar Abbas City, the capital of Hormozgan province, is located on the southern coast of Iran, on the Persian Gulf (56.15–56.42°E and 27.13–27.27°N). In terms of weather conditions, this city has a scorching and humid climate.

### Mosquito collection and rearing

**Fig. 1:**
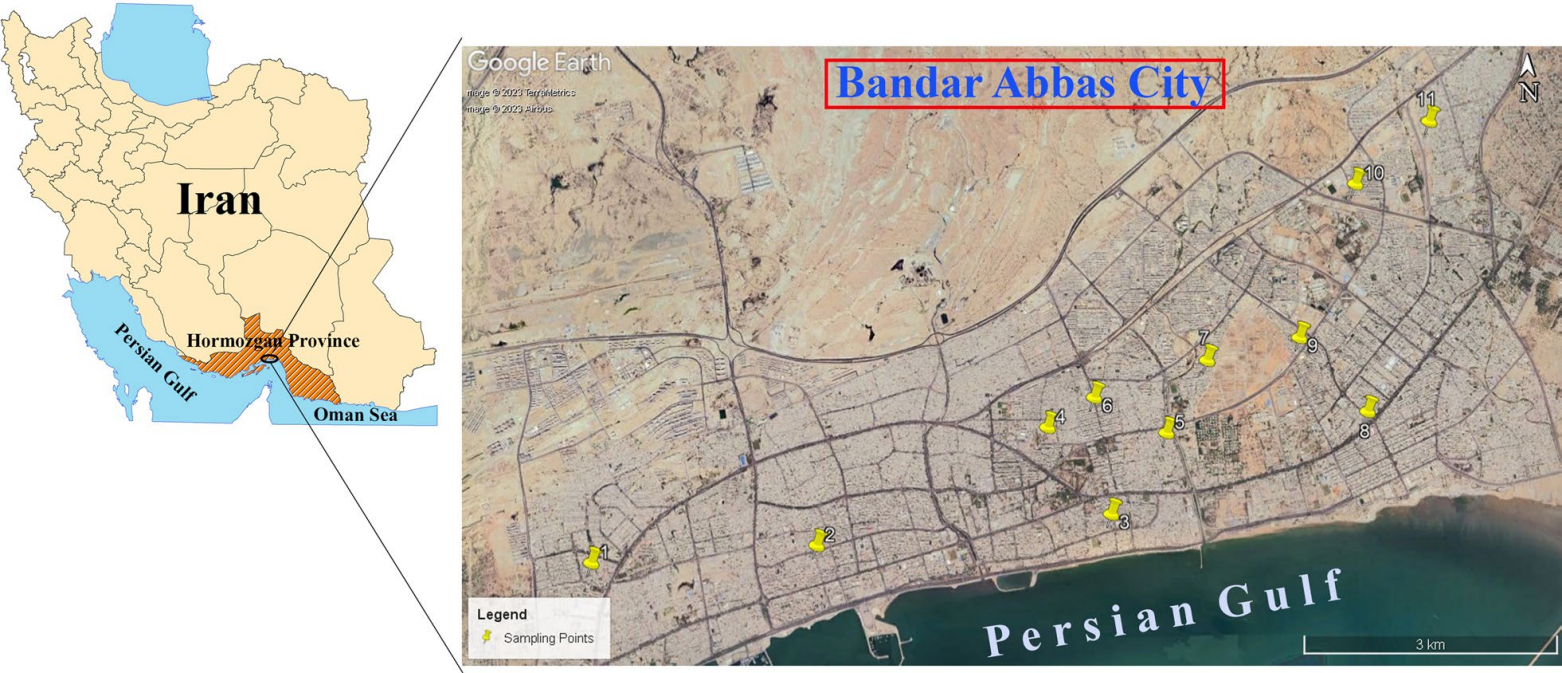
Geographical locations of collection sites. Map was constructed using arc-GIS software, version 10.8 (ESRI, Redlands, CA, USA)

Eggs of *Ae. aegypti* were collected in sticky ovitraps between May and July 2022 in 11 urban localities within Bandar Abbas City (Fig. 1). Details on the geographical properties of these egg collection sites and the respective egg counts are presented in Table 1.

**Table 1:** Geographic characteristics of *Aedes aegypti* egg collection sites detailing the number of eggs collected at each location

Map codes for sampling sites	Sampling sites	Geographic coordination (DD)		Altitude (m a.s.l.)	Collected eggs (*n*)	Eggs analyzed by PCR (*n*)	Eggs included in sequencing analysis (*n*)	Accession number in GenBank
		Latitude (N)	Longitude (E)					
1	Sheshsad Dastgah quarter	27.182°	56.245°	10	107	3	1	OQ997236
2	City center quarter	27.187°	56.273°	7	92	3	1	OQ997237
3	Khaje Ata quarter	27.185°	56.308°	6	196	3	1	OQ997238
4	North Farhangian quarter	27.194°	56.301°	15	132	3	1	OQ997239
5	Seyed Mozafar quarter	27.193°	56.315°	8	246	3	1	OQ997240
6	Azadegan quarter	27.198°	56.306°	19	248	3	1	OR398783
7	Valiasr quarter	27.200°	56.319°	16	114	3	1	OR398784
8	Resalat quarter	27.196°	56.339°	11	95	3	1	OR398785
9	Pardis’s crossroad	27.204°	56.331°	29	143	3	1	OR398786
10	Elahiye quarter	27.220°	56.337°	46	0	0	–	–
11	Tohid quarter	27.227°	56.346°	38	92	3	1	OR398787
	Total				1465	30	10	

*DD* Decimal Degrees

The ovitraps were placed indoors and outdoors in shaded areas to avoid direct sunlight and rain. At each study site, 10 ovitraps were deployed for sampling; at 4-day intervals all ovitraps were replaced with new ones, and the collected ovitraps were transferred to the mosquito insectary at the Hormozgan Medical Sciences, Iran. Larvae emerging from the collected eggs were fed with dry fish food and kept at 26 ± 2 °C and 65% ± 5% relative humidity under a light:dark cycle of 12:12 h. The reared mosquitoes were identified using available morphological keys [41–43] and then stored in a microtube containing ethanol at − 20 °C for molecular tests.

### Molecular tests

The Collins extraction method was utilized to extract DNA from the stored *Ae. aegypti* mosquitoes [44]. A total of 1465 eggs were collected in this study and reared in the laboratory, and three egg samples from each site were randomly selected for PCR analysis, with the exception of one site where no samples were collected. Thus, a total of 30 samples were included in the PCR analysis. For detection of the origin of the *Ae. Aegypti,* we used a 721-bp region of the mitochondrial DNA-encoded cytochrome* c* oxidase subunit I (mtDNA-COI) gene amplified in a thermal cycler using forward primers (GGTCAACAAATCATAAAGATATTGG) and reverse primers (TAAACTTCAGGGTGACCAAAAAATCA) [45].

The PCR reaction was carried out in a total reaction volume of 20 μl (4 mM of MgCl_2_, 1.5 μM of forward primer, 1.5 of reverse primer, 2 mM buffer, 150 mM of each dNTP, 1 U Taq DNA polymerase, 40 ng of DNA and deionized water to correct volume). The thermal conditions for the PCR reaction consisted of an initial denaturation at 95 °C for 10 min; followed by 30 cycles of amplification (denaturation at 95 °C for 1 min, annealing at 54 °C for 1 min and expansion at 70 °C for 1 min; with a final extension at 70 °C for 10 min [46].

### Genetic analysis

Products from the PCR analysis were separated by gel electrophoresis and the bands observed in a gel documentation system. A 100-bp DNA ladder was used as the molecular weight marker. One high-quality 721-bp mtDNA-COI gene fragment from each collection site (10 samples in total) was sequenced by the Livogen Pharmed Company, Tehran, Iran. The sequences were aligned with Clustal W [47], and then edited using the BioEdit sequence analysis tool [48].

The number of haplotypes was computed using the DNAsp software package [49]. Other parameters, including haplotype diversity (Hd), nucleotide diversity (p), the average number of pairwise nucleotide differences and the number of synonymous and non-synonymous mutations, were also calculated using DNAsp software [50–52].

Phylogenetic relationships were explored using MEGA_6_ and BioEdit software [53, 54]. Ultimately, the sequences were recorded in the GenBank database (NCBI).

### Population expansion

To examine neutral mutation, we determined Tajima’s D, Fu and Li’s D^+^ and F^+^ and R_2_ statistics using DNAsp software [49, 51]. Tajima's D was calculated based on the number of different sites. Fu’s Fs statistic was used to assess the demographic stability [55].

## Results

The basic local alignment search tool (BLAST) analysis [56] confirmed the accurate identification of samples as *Ae. aegypti*, with 100% similarity to GenBank sequences. The PCR product was first sequenced and then edited, following which the edited sequences were deposited in the GenBank under accession numbers OQ997236, OQ997237, OQ997238, OQ997239, OQ997240, OR398783, OR398784, OR398785, OR398786 and OR398787.

Overall, of the 10 samples from Iran in the present study and the 19 sequences retrieved from GenBank, the sequencing study revealed only two haplotypes, with a haplotype diversity and haplotype diversity variance of 0.502 and 0.00157, respectively. Among the 604 nucleotides examined, only one site was found to be non-synonymous, with an average number of pairwise nucleotides and average number of nucleotide differences of 0.502 and 0.50246, respectively.

Nucleotide diversity of the *Ae. aegypti* sequences based on the COΙ gene was found to possess a low value of 0.000832, and the number of segregation sites was 1 (Table 2).

**Table 2:** Genetic variability indices for the *Ae. aegypti* samples from southern Iran

Number of sequences	Number of sites	Number of segregating sites (polymorphic)	π	K_a_	K_b_	Ө_s_	Ө_g_	Number of haplotypes (H)	Haploid diversity (Hd)	Variance of haploid diversity (Vhd)
29	604	1	0.000832	0.502	0.50246	0.00042	0.25464	2	0.502	0.00157

* π* Nucleotide diversity,* K*_*a*_ average number of pairwise nucleotide differences,* K*_*b*_ average number of nucleotide differences,* Ө*_*s*_ theta (per site) from eta,* Ө*_*g*_ theta (per sequence) from eta

The results from the pairwise nucleotide differences test indicated a single polymorphic (segregating) site. Harpending’s raggedness statistic (R_2_: 0.2525) was not significant (*P* > 0.05) across any of the populations of *Ae. aegypti*. The average values for Fu and Li’s D^+^ (0.59850) and F^+^ (0.98452) statistic and for and Fu’s F (1.629) statistic were positive. Fu and Li’s test values were not significant. Strobeck’s S statistic value was 0.487 and Tajima’s D value was 1.53395; both results were not statistically significant (*P* > 0.10) (Table 3).

**Table 3:** Population expansion indices for the *Ae. aegypti* samples from southern Iran

D^+^	F^+^	F_s_	S	D	R_2_
0.59850	0.98452	1.629	0.487	1.533954	0.2525

*D*^*+*^ Fu and Li's D test statistic,* F*^*+*^ Fu and Li's F test statistic,* F*_*S*_ Fu's F_S_ statistic,* S* Strobeck's S statistic,* D* Tajima's D,* R*_*2*_ Harpending's raggedness statistic

Substitution pattern and rates were estimated under the Tamura-Nei model (+G). The gamma parameter was used to model evolutionary rate differences among sites (5 categories, [+G]). Mean evolutionary rates in these categories were 0.00, 0.00, 0.01, 0.26 and 4.73 substitutions per site. The A, T/U, C and G nucleotide frequencies were 28.21%, 38.74%, 17.38% and 15.66%, respectively.

**Fig. 2:**
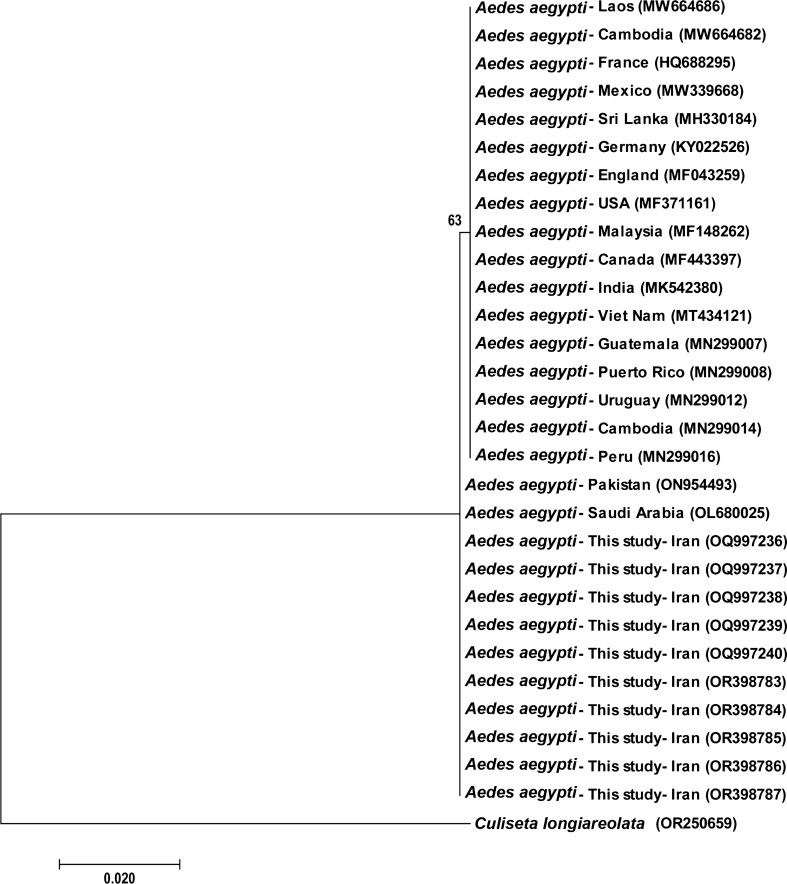
Molecular phylogenetic analysis of the mtDNA-COI gene of *Aedes aegypti* samples and outgroup (*Culiseta longiareolata*) by the maximum likelihood method based on the Tamura-Nei model. The tree with the highest log likelihood (− 1071.43) is shown. The percentage of trees in which the associated taxa clustered together is shown above the branches. The tree is drawn to scale, with branch lengths corresponding to the number of substitutions per site. MtDNA-COI, Mitochondrial DNA-encoded cytochrome* c* oxidase subunit I gene

The phylogeny tree was constructed using the same 29 nucleotide sequences, encompassing 604 nucleotides, from *Ae. aegypti* samples collected in Iran and other countries, based on the mtDNA-COI gene. This tree delineates two primary clades. The first clade includes sequences from samples collected in France, Peru, Uruguay, Puerto Rico, Guatemala, Vietnam, India, Canada, Malaysia, USA, England, Germany, Sri Lanka, Mexico, Laos and Cambodia. Clade 2 includes sequences from samples colleced in Saudi Arabia and Pakistan and Bandar Abbas City in Iran (Fig. 2).

The results indicate a similarity between the mtDNA-COI sequences of *Ae. aegypti* in Hormozgan Province, Iran and those found in Pakistan and Saudi Arabia, as depicted in Fig. 2.

## Discussion

Over the last decade, there has been a significant rise in the prevalence of major arbovirus diseases, such as dengue and Zika. The establishment of *Ae. aegypti*, the primary vector of these diseases, in the southern regions of Iran, spurred our investigation into the population history of *Ae. aegypti* in Iran. Given that the mtDNA marker is a reliable indicator for *Ae. aegypti*, we decided to use COI DNA sequencing data in our study. This gene is extensively employed for the detection of hereditary diversity various mosquito species, including *Ae. aegypti, Aedes vexans, Aedes. caspius, Anopheles gambiae* and *Aedes albopictus* [57, 58]. The results of the present study revealed notably low genetic diversity among the *Ae. aegypti* populations in Iran.

Specifically, our results reveal the presence of two haplotypes that exhibit low diversity (Hd: 0.502) and low nucleotide diversity (π: 0.00083). Generally, low haplotype diversity suggests that the species under study—in this case *Ae. aegypti*—either is not native to the area or has emerged recently [59]. A study in Honduras by Escobar et al. highlighted uniformly low parameters of *Ae. aegypti* genetic diversity, which corroborate the findings of our investigation [1]. On the contrary, the genetic diversity within the *Ae. aegypti* population in Africa stands notably high, strongly suggesting that the African continent is likely *Ae. aegypti*’s place of origin [1, 32]. Comparative assessments have revealed that the genetic diversity of *Ae. aegypti* is lower across all other continents, including Asia, when compared with Africa, implying a subsequent colonization of these continents [32]. Thus, our findings substantiate earlier research outcomes.

Between 1947 and 1970, the insecticide dichlorodiphenyltrichloroethane (DDT) was extensively used in the USA against mosquitoes, specifically targeting *Ae. aegypti*. However, 10 years following the eradication of *Ae. aegypti,* this mosquito was reintroduced into the region, leading to a bottleneck effect within the *Ae. aegypti* population and causing a reduction in their genetic diversity [60].

Changes in host frequency and breeding places among *Ae. aegypti* mosquitoes could potentially further diminish the genetic diversity within populations in the region [58]. In 2018, Joyce et al. collected 84 mosquitoes from various regions across El Salvador and sequenced the COI gene. Their findings revealed an overall haplotype diversity of 0.610 and a nucleotide diversity of 0.016 [61]. Similarly, a study conducted in Panama on 122 mosquitoes from 10 regions reported a nucleotide diversity, haplotype diversity and haplotype diversity of 0.0096, 13 and 0.766, respectively [62]. The results of these two earlier studies indicate a relatively higher genetic diversity within *Ae. aegypti* mosquito populations than was observed in our study, suggesting that it is likely that *Ae. aegypti* has become only recently established in Iran.

The findings of a number of earlier studies using mitochondrial gene markers in *Ae. aegypti* differ from the findings of the present investigation in that the former indicate moderate to higher genetic diversity within their study population. Nonetheless, research carried out across the Asian continent [63–69] indicates a lower genetic diversity in *Ae. aegypti* mosquitoes, consistent with our study outcomes.

This study shows a notably low haplotype diversity within the studied *Ae. aegypti* population, again suggesting the recent establishment of this species in the study area or a limited application of insecticides targeting *Ae. aegypti* [70]. In contrast, Darlina et al. reported a significantly high haplotype diversity in an *Ae. aegypti* population, likely attributable to long-term exposure to insecticides, leading to a high nucleotide mutation rate in the studied population [71]. Such increases in nucleotide mutation rate are generally associated with an increase in resistance within the studied *Ae. aegypti* population [72], possibly linked to extensive use of different groups of insecticides in mosquito control efforts [73]. For example, in regions like Malaysia, the widespread use of synthetic insecticides may be responsible for increases in these types of mutations within mosquito populations [74]. The predictable consequence of this trend is the potential challenge that will need to be met in terms of effectively managing mosquito populations, specifically those of *Ae. Aegypti*, in the future.

A phylogeny tree with two primary branches was constructed using 29 sequences obtained from *Ae. aegypti* samples collected in Iran and various other countries. Samples from Iran, Saudi Arabia and Pakistan clustered within one branch while samples from various countries located on four continents, including France, Peru, Uruguay, Puerto Rico, Guatemala, Vietnam, India, Canada, Malaysia, USA, England, Germany, Sri Lanka, Mexico, Laos and Cambodia, were placed on a separate branch. Consequently, our findings indicate a genetic similarity between the Iranian *Ae. aegypti* population and *Ae. aegypti* samples from Saudi Arabia and Pakistan, suggesting a probable entry of this mosquito into Iran from these countries.

All specimens of *Ae. aegypti* included in this study were placed on the two branches of the phylogeny tree, all the samples used in this study were similar to specimens from other countries, especially Saudi Arabia and Pakistan. Two studies conducted on *Aedes* mosquito populations in India and Thailand reported results exactly the same as our findings, namely that the genetic diversity of *Ae. aegypti* is low across the globe. Taken together, these results suggest that the species' limited genetic diversity may stem from the resemblance of their habitats. This similarity in genetic makeup across habitats might imply recurrent gene flow. However, there is little evidence to support habitat-related genetic diversity [75].

The ratios of G + C and A + T DNA can vary significantly among organisms, and this variation in A + T or G + C content appears to be influenced by mutation pressure, leading to alterations in these nucleotide combinations. Such changes in G + C and A + T content are often associated with substitutions occurring at different positions within codons [76]. Our nucleotide composition analysis in *Ae. aegypti* showed that the presence of the A + T combination was 67%. It has been shown that a relatively high presence of the A + T combination within a species reduces the synonymous position and influences the percentage of amino acid substitutions [76, 77]. Our study demonstrated a low number of synonymous positions owing to the high presence (67%) of the A + T combination in the *Ae. aegypti* population, consistent with previous research [77]. The results of the neutral mutation test on the population of *Ae. aegypti* mosquitoes showed that there has been no population expansion or selection in southern Iran [78]. The outcomes of the neutral mutation test applied to the *Ae. aegypti* mosquito population in southern Iran also indicated no signs of population expansion or selection [64].

Finally, this study confirms by molecular methods that the studied *Ae. aegypti* population exhibits low genetic diversity and that it is highly probable to have entered into southern Iran from Saudi Arabia or Pakistan. To comprehensively understand genetic diversity and analyze gene flow within confined populations of this species, additional mitochondrial genetic markers are imperative. The factors contributing to genetic variation in *Ae. aegypti* could significantly impact its vectorial capacity and competence [65].

## Conclusions

Based on our findings of a low genetic diversity in an Iranian *Ae. aegypti* population, we suggest that this species has been recently established in the southern region of the country. Given the potential impact of genetic variation on the vector's competence for arboviruses, particularly DENV, further studies should be carried out in this field. Our results also suggest that *Ae. aegypti* populations might have entered Iran from Saudi Arabia or Pakistan through transportation associated with international trade. Hence, this possibility should be addressed by the Ministry of Health authorities to prevent the vector's spread to other regions of Iran in the future. Further studies on the genetic diversity of *Ae. aegypti* could significantly contribute to advancing our comprehension of population dynamics, biology and effective management strategies.

## Data Availability

The data used to support the findings of this study are included within the paper.

## Abbreviations

BLAST: Basic local alignment search tool
CHIKV: Chikungunya virus
DENV: Dengue virus
mtDNA-COI: Mitochondrial DNA-encoded cytochrome* c* oxidase subunit I gene
WNV: West Nile virus
YFV: Yellow fever virus
